# Informing the development of services supporting self-care for severe, long term mental health conditions: a mixed method study of community based mental health initiatives in England

**DOI:** 10.1186/1472-6963-12-189

**Published:** 2012-07-07

**Authors:** Gillard Steve, Adams Katie, Edwards Christine, Lucock Mike, Miller Stephen, Simons Lucy, Turner Kati, White Rachel, White Sarah

**Affiliations:** 1 Section of Mental Health, St George’s, University of London, Cranmer Terrace, London, SW17 0RE, UK; 2 NHS Evidence, National Institute for Health and Clinical Excellence, Manchester, UK; 3 Kingston Business School, University of Kingston, London, UK; 4 School of Human and Health Sciences, University of Huddersfield, Huddersfield, UK; 5 South West London & St George’s Mental Health NHS Trust, London, UK; 6 School of Health Sciences, University of Southampton, Southampton, UK; 7 Department of Psychology, Institute of Psychiatry, Kings College London, London, UK

**Keywords:** Long term conditions, Mental health, Self-care, Self-management, Peer support

## Abstract

**Background:**

Supporting self-care is being explored across health care systems internationally as an approach to improving care for long term conditions in the context of ageing populations and economic constraint. UK health policy advocates a range of approaches to supporting self-care, including the application of generic self-management type programmes across conditions. Within mental health, the scope of self-care remains poorly conceptualised and the existing evidence base for supporting self-care is correspondingly disparate. This paper aims to inform the development of support for self-care in mental health by considering how generic self-care policy guidance is implemented in the context of services supporting people with severe, long term mental health problems.

**Methods:**

A mixed method study was undertaken comprising standardised psychosocial measures, questionnaires about health service use and qualitative interviews with 120 new referrals to three contrasting community based initiatives supporting self-care for severe, long term mental health problems, repeated nine months later. A framework approach was taken to qualitative analysis, an exploratory statistical analysis sought to identify possible associations between a range of independent variables and self-care outcomes, and a narrative synthesis brought these analyses together.

**Results:**

Participants reported improvement in self-care outcomes (e.g. greater empowerment; less use of Accident and Emergency services). These changes were not associated with level of engagement with self-care support. Level of engagement was associated with positive collaboration with support staff. Qualitative data described the value of different models of supporting self-care and considered challenges. Synthesis of analyses suggested that timing support for self-care, giving service users control over when and how they accessed support, quality of service user-staff relationships and decision making around medication are important issues in supporting self-care in mental health.

**Conclusions:**

Service delivery components – e.g. peer support groups, personal planning – advocated in generic self-care policy have value when implemented in a mental health context. Support for self-care in mental health should focus on core, mental health specific qualities; issues of control, enabling staff-service user relationships and shared decision making. The broad empirical basis of our research indicates the wider relevance of our findings across mental health settings.

## Background

This paper reports on a multisite, mixed method study which aimed to explore the implementation of generic United Kingdom (UK) policy guidance on supporting self-care for long term conditions in the context of mental health services for people with long term, severe mental health problems [1]. Self-care for long term health conditions has been described as those activities performed independently by an individual to promote and maintain personal health and wellbeing [2]. The need for improved systems to support self-care for long term conditions has been identified internationally, especially where populations are aging and at a time of economic constraint [3,4]. UK health policy advocates the application of a number of generic service delivery components of supporting for self-care across long term conditions, including peer support groups, access to information about services and strategies for self-care, training for personal care planning, and lay-led and community-based delivery [5-7]. In a related policy initiative [8] the Expert Patients Programme – an anglicised adaptation of the Chronic Disease Self-Management Programme [9] – has been widely implemented across UK health care services over the last decade.

UK self-care policy specifies supporting self-care in order to improve outcomes such as confidence, quality of life and satisfaction with services, while reducing use of other health services [5,6]. The evidence base for the effectiveness of interventions supporting self-care across long term conditions is understandably diffuse [10]. Evaluation of the Expert Patients Programme did find some improvement related to quality of life and self-efficacy, although did not show reduction in health care services utilisation [11].

Within mental health, robust evidence for the effectiveness of supporting self-care is limited to trials of Cognitive Behavioural Therapy (CBT) based guided self-help interventions [12]. However guided self-help interventions are recommended as low intensity psychological interventions for mild to moderate common mental health problems [13,14] so may not be effective where the mental health condition is more severe or more persistent. Internationally there is some evidence that generic self-management programmes can improve quality of life for people with long term mental health conditions [15]. More recent evidence on the impact of mental health specific self-management programmes seems to suggest the potential for improvement in health and psychosocial outcomes in addition to quality of life [16]. There is therefore a need for more research on the effectiveness of self-care approaches for people with severe and long term mental health problems.

Enhancing the ability of people experiencing mental health problems to manage their own lives through access to evidence based support has been reprioritised in new UK mental health strategy [17]. However given the diffuse evidence based referred to above it is unclear how this generic self-care policy guidance should best be implemented in mental health services. It has been suggested that service user attitudes to engaging with mental health services might not be the same as in physical health services, and that this might act as a barrier to engagement with support for self-care in mental health [18]. A recent review of qualitative research exploring insight into the nature of self-care from the perspectives of people experiencing mental health problems identified choice, control and engagement as key processes that enable individuals to access appropriate support for self-care [19]. There is some evidence that engagement with mental health services is associated with the quality of the therapeutic relationship between service user and provider at an individual level [20]. The provision of support for self-care represents a shift in that relationship and has been termed a ‘cultural revolution’ [21] 1 and ‘paradigm shift’ [22] for practitioners and services. Organisational change literature suggests that role change of this sort brings about challenges over professional task and expertise boundaries shaped by a range of factors including client support [23]. It has been argued that consensus about role expectation enables meaningful adoption of changed roles within teams [24] and research into the self-management of long term conditions has highlighted discrepancies between staff and service user expectations [25]. Understanding service user perspectives on new roles and relationships with staff supporting self-care, as well as issues of control and engagement, is therefore a crucial element of exploring implementation issues around supporting self-care in mental health services.

The review of qualitative self-care research cited above revealed a lack of conceptual clarity around the term self-care in the mental health context, suggesting that self-care encompasses related concepts of self-management, self-help and recovery [19]. It has been suggested that this lack of conceptual distinctiveness, together with the disparate nature of the evidence base act as barriers to providing self-care support [26]. Similarly, as result of the disparate nature of current provision and a lack of clear empirical indication of what constitutes self-care outcomes it is difficult to design a controlled study. In order to address those challenges service providers need empirical insight into how a range of mental health specific implementation variables are likely to be associated with outcomes. This evidence is most efficiently developed in an observational study that undertakes exploratory analyses of what, according to current policy, practice and the existing, limited evidence base, are likely to be relevant variables. The aims of this paper are therefore to inform the development of effective self-care support in a mental health context by:

1) Identifying whether service delivery components advocated in generic self-care policy are associated with self-care outcomes;

2) Identifying and understanding how mental health specific implementation issues (e.g. choice, control, engagement, staff-service user relationship) impact on the provision of support for self-care in mental health;

3) Considering whether and how these issues apply across mental health services or are specific to particular settings or populations.

**Figure 1:**
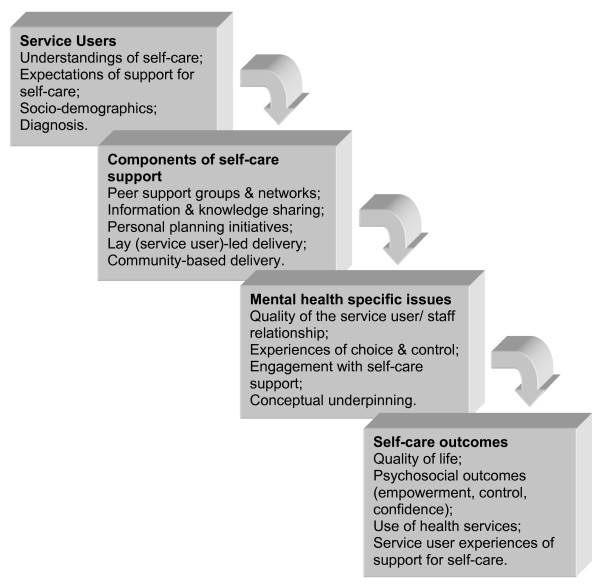
Framework for investigating support for self-care in mental health.

The literature presented above has suggested a number of service user level issues that might inform the provision of support for self-care in mental health. We present these as a framework for investigation in Figure 1 below.

## Method

### Study design

A mixed method study design was employed in order to address the research aims identified above, comprising a cohort study of 120 consecutive new, adult referrals to three contrasting initiatives supporting mental health self-care, and qualitative interviews with cohort study participants at both baseline and follow up. Given that an evidence base is already emerging for guided self-help and self-management approaches to supporting self-care, as cited above, we selected as our case studies community based initiatives that sought to support self-care for people with severe, long term mental health problems. A mixed method approach was used because research aims would be best addressed through both qualitative analysis of service user accounts of their experiences of support for self-care, and through a quantitative approach that sought to explore statistical associations between a range of implementation and outcome variables. A comparative case study approach was also integrated into the study design – sampling from three, contrasting initiatives rather than a single, homogeneous population – in order that we could consider the extent to which our observations applied across mental health settings (aim 3). The implications of sampling from contrasting populations are considered in the Discussion section below.

### Setting

The setting for this study was Mental Health NHS Trusts in southern England (SO), London (LO) and northern England (NO) situated in a range of urban/rural and socio demographic areas. We noted above an overlap between the concepts of self-care, recovery and self-management. When selecting sites for this study we found a range of initiatives that were described as providing support for self-care but which explicitly referred to other, related concepts in their theoretical underpinning. We selected sites purposively on the basis of the following criteria:

1. Initiatives comprised of service delivery components advocated in self-care policy guidance;

2. Initiatives that sought to improve outcomes identified in self-care policy guidance;

3. Initiatives underpinned by concepts identified in the wider literature as relating to self-care.

Candidate sites were identified through the professional and research networks of the research team. Detailed discussions were had with clinical leads at each site to ensure that initiatives met inclusion criteria. Sites were selected by the research team to provide variation across criteria and so inform our exploratory analyses.

The initiative in southern England (SO) was informed by a recovery approach; recovery has become a guiding principle as mental health services seek to empower service users to take more control of their own lives [18]. Mental health professional and service user facilitators trained groups of people who used services in the Mental Health Trust to develop and use an eight section personal and crisis plan over a ten week course; Wellness Recovery Action Planning (WRAP) [27].

The London initiative (LO) was an open access peer support group project for people who identified as experiencing personality disorders (i.e. they did not need to have a formal diagnosis of Personality Disorder) co-facilitated by trained service users and professionals from the Mental Health Trust. Group process was informed by Coping Process Theory [28] – group members use the group to appraise their perceptions of threat to the self and to develop coping strategies – and groups took place in community locations outside of the Mental Health Trust to reduce the experience of stigma that group members might associate with having attended Personality Disorders services in the past.

The northern England initiative (NO) was a community arts project provided by a voluntary sector agency and jointly commissioned by the Mental Health Trust and Local Authority Social Services Department. The Mental Health Trust signposted people using mental health services to the project. This project was informed by a social inclusion perspective that suggests that closeness to mainstream society can support an individual’s personal recovery, improve their sense of empowerment and enable them to reclaim control and responsibility for themselves [29].

### Participants

Participants were 121 adults using secondary mental health services consecutively referred to the initiatives supporting self-care (inclusion criteria for the study were those of the participating services) recruited over a nine month period. Participants were excluded if they were considered by staff to be too unwell to participate in interviews. Potential participants were identified by team leaders at each site who introduced the study and participant information to them. If they were interested in participating they were contacted by a member of the research team who met them and, if they remained interested, obtained their informed consent to participate. The study, including the recruitment process, was reviewed and approved by the UK NHS Research Ethics Committee London-London Bridge. Data will be stored at the first author’s institution and made available for legitimate research purposes as detailed in the UK Medical Research Council’s policy on sharing of research data from population and patient studies.

### The quantitative study

A cohort study design enabled us to conduct exploratory analyses to identify the extent to which a range of implementation variables were associated with change in self-care outcomes. For example, we were able to consider the extent to which participant engagement with specific components of the initiatives supporting self-care (e.g. attendance of peer support groups or completion of personal plans), or service user-staff relationships were associated with change in outcomes. Together with qualitative findings, this would enable us to understand how different approaches to supporting self-care might be applied within and across mental health settings.

The outcomes explored were quality of life, empowerment and mental health confidence, as well as use of mental health services in the previous nine months. Selection of outcomes was guided by the literature cited above.

Outcome measurements were made at baseline and at 9 months follow up by means of the following self report tools:

"Schedule for the Evaluation of Individual Quality of Life – Direct Weighted version (SEIQoL-DW) [30];"

"User Empowerment Measure [31] – a ‘consumer developed’ scale that conceptualises empowerment as self-esteem/self-efficacy, feelings of power and consumer activism;"

"Mental Health Confidence Scale [32] – a measure of the confidence people experiencing mental distress have in their own ability to deal with things that commonly influence their lives, with confidence is conceptualised in terms of optimism, coping and (self)advocacy;"

"Service use for the 9 months prior to baseline and the 9 month exposure period was measured using a modified version of the Client Socio-demographic and Service Receipt Inventory (CSSRI) [33] – this data was not used for economic analyses."

Demographic, diagnosis and psychiatric history data were collected at baseline using the self-report CSSRI tool. Clinical severity at baseline was measured using the Clinical Outcomes in Routine Evaluation - Outcome Measure (CORE-OM) [34], comprising four subscales of wellbeing, symptoms, functioning and risk. A non-risk score is derived from the three non-risk subscales. Higher scores indicate greater clinical severity.

At 9 month follow up experience of therapeutic relationship was measured by asking the participant to complete the Scale to Assess Therapeutic Relationships in Community Mental Health Care (STAR) (patient version) [35] about the member of intervention staff with whom they had the most contact in the preceding 9 months. This scale has three subscales: positive collaboration; positive clinician input; non supportive clinician. Higher scores indicate a better therapeutic relationship.

In the absence of a standardised measure of engagement with components of complex interventions, it was necessary to develop a measure that was tailored to each intervention [36] that indicated either a higher or lower level of engagement. Through consultation with delivery teams at each site ‘higher engagement’ was indicated as follows: SO - partially or fully completed WRAP plans during training sessions AND continued to work on the plans after training sessions had finished; LO - still attending peer support group at the time of follow up interview OR expressed an intent to return after a break in attendance; NO - attended at least 60% of possible sessions.

### Calculation of sample size

For the purposes of an exploratory analysis of this sort it was calculated that recruiting 32 participants in each site would enable meaningful change in the range of outcomes, a within site medium effect size = 0.49, to be detected with 80% power at a 5% significance level. For example, assuming a baseline standard deviation of Empowerment equal to 10.7 - as found in a sample of Community Mental Health Team service users with psychotic disorders [37] - a within site effect size of 0.49 equates to a change in Empowerment of 5.2. To allow for a realistic 20% attrition of the sample between baseline and 9 month follow up, we aimed to recruit 40 service users at each site.

### Statistical analysis strategy

A statistical analysis strategy was designed to enable us to investigate study aims by exploring associations between a range of implementation variables and outcomes, specified a priori as suggested by the literature. For comparison between those lost to follow up and completers, two samples *t*-test or Mann Whitney U Tests for continuous baseline characteristics (dependent on distribution) and *χ*^2^ tests for categorical variables were employed. Comparison between sites was conducted using one-way analysis of variance or Kruskal-Wallis Tests for continuous baseline characteristics (dependent on distribution) and *χ*^2^ tests for categorical variables. No imputation of missing data was conducted. Subsequent detailed analysis proceeded in three distinct stages.

#### Change in outcomes

Continuous outcomes, which were normally distributed, were compared between baseline and 9 month follow up by the calculation of the mean difference and 95% confidence intervals. Effect sizes (ES) were calculated by dividing the mean difference by the baseline standard deviation. Discrete variables were compared between baseline and 9 month follow up using the Wilcoxon signed ranks test. For ease of presentation these discrete variables have been categorised into appropriate intervals and the count and percentage of patients falling into each interval is presented. These analyses have been conducted within each site and overall.

#### Variables associated with outcomes

In this analysis three dependent variables were analysed: quality of life; empowerment; mental health confidence. The following baseline variables were tested for a univariate association with the dependent variables: age; gender; marital status; highest education achieved; living situation; accommodation status; employment status; site; on typical antipsychotics; on atypical antipsychotics; on mood stabilisers; on anti-depressants; on depot injections; number of psychotropic medications; concordance; excessive or problem drinking; problem drug use; chronicity; number of lifetime psychiatric admissions; CORE Well being score; CORE Problems and symptoms score; CORE Functioning score; CORE Risk score; CORE Non-risk score; CORE Clinical score; STAR Overall score; engagement with the self-care intervention.

Analysis of covariance was used to test for associations between each of the above variables and the dependent variable, while controlling for the baseline level of the dependent variable. Variables found to be univariately associated with the dependent variable at the 10% significance level were retained for consideration for entry into a final model. Before conducting the final model, an assessment of whether these retained variables were independent of each other was made. Where variables were significantly associated, a decision would be made as to the most appropriate variables to enter simultaneously into the final model.

#### Predictors of engagement

The dependent variable in this analysis indicated whether engagement with the self-care intervention had been higher or lower, as defined for each site. Independent variables tested for possible association with engaging were as above but also included quality of life, empowerment, mental health confidence, all at baseline and STAR Overall score and the three STAR subscales, all rated at follow up. Logistic regression was used and each independent variable tested univariately for an association with engagement. Analysis then proceeded as above.

### The qualitative study

Our qualitative study sought to identify how different service delivery components - as well as issues around choice, control and relationships - supported self-care from the service user perspective. We felt it was necessary first to elicit understandings of self-care and expectations of self-care support. At baseline a 45 minute, semi-structured interview was conducted with all participants, digitally recorded and transcribed verbatim. A similar interview at nine months follow-up explored participants’ experiences of the intervention and their understandings of how support for self-care had impacted on their wider lives. Development of interview schedules was informed by: (1) existing policy and research literature about self-care; (2) the experiences of clinical members of the research team of providing mental health services; (3) the experiences of service user members of the research team, and their understandings and expectations of support for self-care. Schedules were piloted with people using services similar to those included in the study and questions amended to address the accessibility and relevance of questions.

#### Qualitative analysis

The analysis of qualitative data proceeded in three stages: (i) development of an organising framework for analysing interview transcripts; (ii) synthesis of qualitative data with quantitative findings; (iii) articulation of overarching themes that provided insight and explanation of how self-care is supported in mental health.

The organising framework was developed through, firstly, a preliminary thematic analysis of a subsample of interview transcripts from each site using coding tools common to inductive thematic analysis [38]. Secondly, a matrix approach [39] enabled comparison of emerging themes across the three sites and the iterative development of a thematic framework that could be applied to the whole qualitative dataset. Finally, this framework was used to code the entire dataset to an NVivo database. As coding proceeded flexibility was retained in order that new themes could be developed where data did not fit into existing themes. Researchers at different sites regularly cross-checked their coding of subsamples of the data. Where there were discrepancies these were discussed as a team and if necessary the content and boundaries of themes revised.

In the second stage of the qualitative analysis we followed accepted principles of narrative data synthesis [40,41], identifying where: (a) triangulation across datasets enables qualitative data to be used to further elucidate quantitative analysis; (b) tensions between datasets articulates complexities in the findings that can inform refining of interventions. To do this a number of systematic ‘queries’ were asked of the qualitative data, derived from the results of the statistical analysis. NVivo qualitative analysis software was used to assign ‘attributes’ to individual interview transcripts in the qualitative database (for example: higher or lower level of engagement; whether the participant reported taking their medication as prescribed or not). We then cross-tabulated these attributes with qualitative data coded to particular themes (for example data coded to the themes ‘relationships with staff’ or ‘medication’). This produced a series of ‘query reports’ that we used to explore in depth the insight offered into statistical analyses by our qualitative data. The third stage sought to add an interpretative layer of meaning to the data [42] by identifying overarching themes that would explain and provide insight into mental health specific issues of supporting self-care.

## Results

### Participants

**Figure 2:**
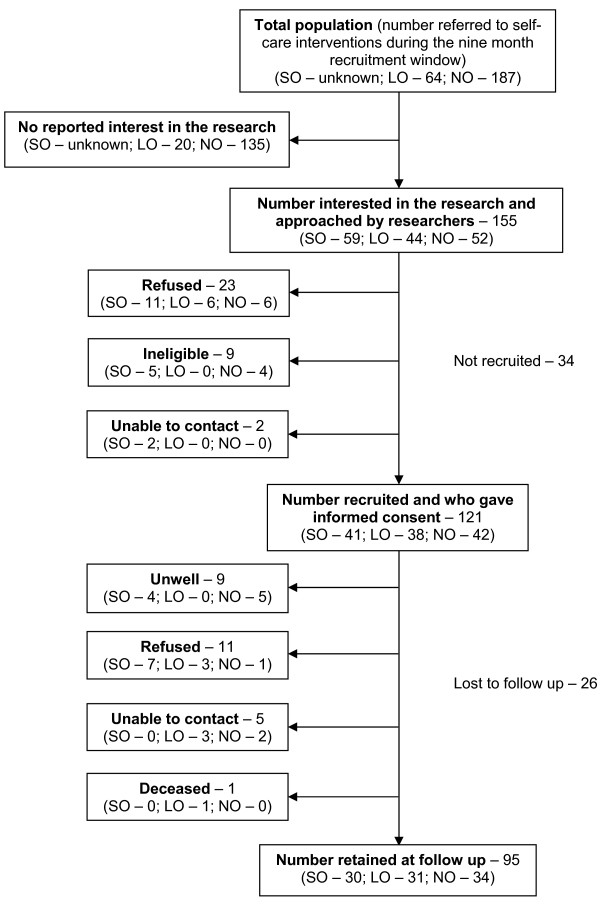
**Participants in the study.** In the SO site the intervention was provided by training members of Trust staff and service users to deliver the intervention. 228 people were trained to deliver the intervention during the recruitment window. It was not possible to quantify how many of those trainers then made the intervention available to how many people using the services they worked in.

The flow of participants through the study is indicated in Figure 2.

### Descriptive data

A description of the sample is given in Table 1 below. The main points of comparison between the sites can be summarised as follows. LO had the youngest sample with a higher proportion of single people (74%) and a more ethnically diverse sample. SO had the lowest rate of unemployment (49%) and NO had the higher number of dependent children. There was also a significant variation in admissions to hospital in the previous 9 months across the sites with the highest proportion in SO (51%). A higher proportion of participants (45%) used A&E for psychiatric reasons in LO. LO had the highest proportion of participants (42%) who reported personality disorder as their primary diagnosis. SO and NO had similar diagnostic case mix. Rates of harmful drinking and drug use varied across the sites with LO having the highest proportion of both these variables.

**Table 1:** Baseline characteristics and comparisons between sites

**Variable**	**Label**	**LO (peer groups) (n = 38)**	**SO (personal plans) n = 41)**	**NO (community arts) (n = 42)**	**Total (n = 121)**	**Significance**
**Demographics**						
**Age**	**Mean (SD)**	36.3 (10.8)	44.2 (12.5)	43.7 (10.7)	41.5 (11.8)	F (2, 117) = 5.9,
	**Min – Max**	18 – 61	24 – 64	19 – 65	18 – 65	p = .003
**Gender**	**Female**	28 (74%)	28 (68%)	26 (62%)	82 (68%)	*X*^2^ = 1.3, df = 2, p = .529
**Ethnic group**	**White-British**	24 (63%)	41 (100%)	36 (86%)	101 (84%)	*x*^2^ = 20.3, df = 6, p = .002
	**White-Other**	10 (26%)		5 (12%)	15 (12%)	
	**Other**	3 (8%)		1 (2%)	4 (3%)	
**No. dependents**	**0**	27 (71%)	36 (88%)	27 (64%)	90 (74%)	Kruskall Wallis
	**1**	4 (11%)	3 (7%)	6 (14%)	13 (11%)	*x*^2^ = 6.7, df = 2, p = .036
	**2**	3 (8%)	1 (2%)	4 (10%)	8 (7%)	
	**3 +**	4 (11%)	1 (2%)	5 (12%)	10 (8%)	
**Marital status**	**Single**	28 (74%)	17 (42%)	22 (52%)	67 (55%)	
	**Married/co**	3 (8%)	11 (27%)	11 (26%)	25 (21%)	*x*^2^ = 9.8, df = 4, p = .044
	**Sep/div**	7 (18%)	13 (32%)	9 (21%)	29 (24%)	
**Highest education**	**GCSE**	16 (42%)	22 (54%)	24 (57%)	62 (51%)	*x*^2^ = 6.4, df = 4, p = .172
	**Above GCSE**	22 (58%)	19 (46%)	16 (38%)	57 (47%)	
**Living situation**	**Living alone**	25 (66%)	20 (49%)	24 (57%)	69 (57%)	*x*^2^ = 2.3, df = 2, p = .312
**Accommodation**	**Supported accom**	24 (63%)	24 (59%)	24 (57%)	72 (60%)	*x*^2^ = .3, df = 2, p = .851
**Employment status**	**Unemployed**	27 (71%)	20 (49%)	31 (74%)	78 (65%)	*x*^2^ = 6.7, df = 2, p = .035
**Service Use**						
**Psychiatric admission in previous 9 months**	**Yes**	9 (24%)	21 (51%)	12 (29%)	42 (35%)	*x*^2^ = 7.7, df = 2, p = .022
**Attended A&E for psychiatric reason in previous 9 months**	**Yes**	17 (45%)	2 (5%)	2 (5%)	21 (17%)	*x*^2^ = 18.1, df = 2, p < .001
**Used Crisis/Home Treatment team in previous 9 months**	**Yes**	8 (21%)	7 (17%)	8 (19%)	23 (19%)	*x*^2^ = .4, df = 2, p = .814
**Psychiatric history**						
**Number of life-time psychiatric admissions**	**Never**	17 (45%)	10 (25%)	12 (29%)	39 (33%)	*x*^2^ = 0.1, df = 8, p = .173
	**1 – 2**	8 (21%)	11 (28%)	8 (19%)	27 (23%)	
	**3 – 5**	6 (16%)	7 (18%)	15 (36%)	28 (23%)	
	**6 – 10**	5 (13%)	9 (23%)	3 (7%)	17 (14%)	
	**11+**	2 (5%)	3 (8%)	4 (10%)	9 (8%)	
**Time since first contact with services (years)**	**Mean (SD)**	13.8 (9.6)	16.6 (13.0)	13.6 (10.2)	14.7 (11.0)	F (2, 117) = .9, p = .401
	**Min – Max**	.25 – 40	.25 – 46	.08 – 37	.08 – 46	
**Primary Diagnosis**	**Personality disorder**	16 (42%)	4 (10%)	1 (2%)	21 (17%)	*x*^2^ =39.9, df = 10, p = .001
	**Schizophrenia**	1 (3%)	12 (29%)	7 (17%)	20 (17%)	
	**Bipolar**	2 (5%)	4 (10%)	8 (19%)	14 (12%)	
	**Anxiety/depression**	16 (42%)	18 (44%)	17 (41%)	51 (42%)	
	**Other**	1 (3%)	2 (5%)	7 (17%)	10 (8%)	
	**Not known**	0 (0%)	0 (0%)	1 (2%)	1 (1%)	
**Number of psychotropic medications**	**Mean (SD)**				2.4	F (2, 118) = .3, p = .749
	**Min – Max**	2.2 (1.6)	2.4 (1.6)	2.5 (1.2)	(1.2)	
		0 – 5	0 – 6	1 – 5	0 – 6	
**On typical anti-psychotics**	**Yes**	4 (11%)	9 (22%)	6 (14%)	19 (16%)	*x*^2^ = 2.0, df = 2, p = .360
**On atypical anti-psychotics**	**Yes**	10 (26%)	20 (49%)	18 (43%)	48 (40%)	*x*^2^ = 4.4, df = 2, p = .109
**On mood stabilisers**	**Yes**	2 (5%)	6 (15%)	5 (12%)	13 (11%)	*x*^2^ = 1.9, df = 2, p = .387
**Anti-depressant**	**Yes**	30 (79%)	26 (63%)	35 (83%)	91 (75%)	*x*^2^ = 4.8, df = 2, p = .089
**Depot injections**	**Yes**	1 (3%)	7 (17%)	1 (2%)	9 (7%)	*x*^2^ = 8.4, df = 2, p = .015
**Medication taken as prescribed**	**Yes**	23 (62%)	34 (83%)	30 (71%)	87 (72%)	*x*^2^ = 9.4, df = 6, p = .153
	**Partially**	11 (29%)	5 (12%)	9 (21%)	5 (21%)	
	**No**	1 (3%)	1 (2%)	3 (7%)	5 (4%)	
**Alcohol use**	**No problem**	21 (55%)	36 (88%)	33 (79%)	90 (74%)	*x*^2^ = 26.0, df = 6, p < .001
	**At risk**	9 (24%)	2 (5%)	9 (21%)	20 (17%)	
	**Harmful**	8 (21%)	1 (2%)	0	9 (7%)	
**Drug use**	**No problem**	30 (79%)	37 (90%)	40 (95%)	107 (88%)	*x*^2^ = 12.7, df = 6, p = .048
	**Receiving help**	6 (16%)	2 (5%)	2 (5%)	10 (8%)	
	**Harmful**	2 (5%)	0	0	2 (2%)	
**Psychological measures**						
**Quality of Life**	**Mean (SD)**	46.7 (20.8)	58.1 (23.6)	61.2 (21.4)	55.6 (22.7)	(F (2, 117) = 4.8, p = .010),
	**Min – Max**	9 – 85	12 – 100	14 – 95	9 – 100	
**Empowerment**	**Mean (SD)**	69.3 (8.8)	74.6 (12.9)	74.0 (11.3)	72.7 (11.3)	(F (2, 117) = 2.6, p = .081)
	**Min – Max**	52 – 90	33 – 106	41 – 96	33 – 106	
**Mental Health Confidence**	**Mean (SD)**	3.0 (1.0)	3.6 (1.0)	3.5 (.9)	3.37 (1.0)	(F (2, 117) = 5.0, p = .008),
	**Min – Max**	1 – 6	2 – 5	2 – 5	1 – 6	
**CORE wellbeing**	**Mean (SD)**	19.5 (3.9)	18.2 (5.0)	20.3 (5.4)	19.3 (4.9)	(F (2, 118) = 1.9, p = .148)
	**Min – Max**	10 – 28	10 – 35	10 – 33	10 – 35	
**CORE problems and symptoms**	**Mean (SD)**	28.3 (8.1)	16.5 (8.8)	21.2 (9.4)	21.8 (10.0)	(F (2, 118) = 17.9, p < .001),
	**Min – Max**	7 – 38	2 – 33	3 – 38	2 – 38	
**CORE functioning**	**Mean (SD)**	19.8 (3.8)	17.8 (3.1)	19.1 (4.4)	18.9 (3.9)	(F (2, 118) = 2.9, p = .059)
	**Min – Max**	11 – 29	11 – 25	6 – 28	6 – 29	
**CORE risk**	**Mean (SD)**	14.2 (8.3)	5.3 (7.5)	7.3 (7.8)	8.9 (8.7)	(F (2, 118) = 14.6, p < .001)
	**Min – Max**	0 – 30	0 – 25	0 – 28	0 – 30	
**CORE non-risk**	**Mean (SD)**	23.3 (4.5)	17.1 (4.7)	19.8 (5.3)	20.0 (5.4)	(F (2, 118) = 16.3, p < .001)
	**Min – Max**	12 – 32	10 – 28	11 – 33	10 – 33	
**CORE clinical score**	**Mean (SD)**	21.7 (4.6)	15.2 (4.7)	17. 8 (5.3)	18.1 (5.5)	(F (2, 118) = 18.4, p < .001)
	**Min – Max**	10 – 31	8 – 27	9 – 31	8 – 31	

LO had the highest CORE clinical score (mean: 21.7, SD: 4.5) and scored the highest on CORE subscales problems and symptoms, functioning, risk, and non-risk compared to the other sites. NO had the highest mean Quality of Life score and LO the lowest. LO had a lower mean score on Mental Health Confidence than the other two sites but the sites did not differ in terms of Empowerment.

### Lost to follow up

Participants who were not interviewed at follow up (n = 26) had been in contact with services for a shorter period of time (p = 0.030); they were on less medication (p = 0.038); they scored lower on CORE clinical scores (p = 0.003) and CORE non risk scores (p = 0.040); they scored higher on the CORE risk scores (p = 0.027). However after a Bonferroni correction for multiple testing these differences were no longer significant. The impact of medication on outcome is considered in detail below.

### Results - quantitative study

#### Change in outcomes

Average length of follow up was 8.9 months, 82% had their follow up interview within an 8 to 10 month window. Changes in outcome measures are detailed in Table 2 below. Improvement was found in the total sample in quality of life, empowerment and mental health confidence, with effect sizes of 0.25, 0.26 and 0.32 respectively. In the LO site effect sizes of 0.41 and 0.5 were found in empowerment and mental health confidence, respectively. No statistically significant improvement was found in quality of life. An improvement in mental health confidence was found in the NO site with an effect size of 0.31, with no significant change found in the other measures.

**Table 2:** Overall and within site changes in outcome measures

	**n**	**T0**^**a**^	**T1**^**a**^	**Change**^**b**^	**ES**
**Quality of Life**					
**LO**	31	47.9	54.4	−6.5	0.30
		(21.5)	(20.5)	(−14.6, 1.5)	
**SO**	26	58.2	60.9	−2.7	0.11
		(25.2)	(22.9)	(−16.5, 11.0)	
**NO**	34	61.0	68.1	−7.1	0.35
		(20.5)	(18.0)	(−14.9, 0.8)	
**Total**	91	55.7	63.4	−5.6	0.25
		(22.7)	(20.9)	(−11.1, -0.2)	
**Empowerment**					
**LO**	31	70.0	73.9	−3.9	0.41
		(9.4)	(9.3)	(−7.0, -0.8)	
**SO**	28	73.2	76.2	−3.0	0.22
		(13.9)	(9.4)	(−6.4, 0.4)	
**NO**	34	72.6	74.9	−2.2	0.19
		(11.7)	(9.7)	(−5.7, 1.2)	
**Total**	93	71.5	75.0	−3.0	0.26
		(11.7)	(9.4)	(−4.9, -1.2)	
	**Mental Health Confidence**				
**LO**	31	3.08	3.58	−0.50	0.5
		(1.0)	(0.9)	(−0.78, -0.22)	
**SO**	28	3.58	3.77	−0.19	0.17
		(1.1)	(1.0)	(−0.55, 0.17)	
**NO**	34	3.39	3.67	−0.28	0.31
		(0.9)	(0.9)	(−0.56, 0.00)	
**Total**	93	3.35	3.67	−0.32	0.32
		(1.0)	(0.9)	(−0.50, -0.15)	

^a^Data is mean and (standard deviation).

^b^Data is mean difference and (95% confidence intervals).

Overall, there was a decrease in the number of psychiatric A&E attendances (p = 0.007), also significant within the LO site (p = 0.005), shown in Table 3 below.

**Table 3:** Overall and within site change in use of A&E for psychiatric emergency

		** *Number of psychiatric A&E attendances (in previous 9 months)* **						
		**T0**			**T1**			**p-value**^**a**^
	**n**	**0**	**1 - 5**	**6 - 10**	**0**	**1 - 5**	**6 - 10**	
**LO**	**31**	17	11	3	28	2	1	0.005
		(55%)	(35%)	(10%)	(90%)	(7%)	(3%)	
**SO**	**26**	25	1	0	26	0	0	0.317
		(96%)	(4%)	(0%)	(100%)	(0%)	(0%)	
**NO**	**33**	31	2	0	31	2	0	0.705
		(94%)	(6%)	(0%)	(94%)	(6%)	(0%)	
**Overall**	**90**	73	14	3	85	4	1	0.007
		(81%)	(16%)	(3%)	(94%)	(5%)	(1%)	

^a^ Wilcoxon signed ranks test.

#### Variables associated with outcomes

Being on typical antipsychotics, on depot injections, the number of psychotropic medications, CORE Non-risk, CORE Wellbeing and CORE Problems and Symptoms scores (all as measured at T0) were all univariately associated with quality of life (SEIQoL-DW) at follow up at the 10% level. After removing highly correlated variables the retained variables - number of psychotropic medications, CORE-non risk score - were entered into an ANCOVA. In the combined model it was found that a higher number of psychotropic medications (B = 2.7: 95% CI -.14, 5.5) and a lower CORE Non-risk score at T0 (B = −.83: 95% CI −1.6, -.05) were both associated with higher quality of life at 9 months follow up.

Number of psychotropic medications and choosing to take medication as prescribed (as measured at T0) were associated with Empowerment at 9 months follow up at the 10% level. It was found that a lower number of psychotropic medications (B = −1.1: 95% CI - 2.2, -.13) and taking medication as prescribed (Yes; Mean = 76.4: 95% CI 74.7, 78.0, No; Mean = 71.2: 95% CI 65.2, 77.1) at T0 were associated with higher empowerment at follow up.

Choosing to partially take medication as prescribed at baseline was associated with lower mental health confidence at follow up (Mean = 3.4: 95% CI 3.1, 3.7). These findings are shown in Table 4 below.

**Table 4:** Association between baseline variables and outcomes at follow up

**Variable**		** *B o Mean(95% CI)* **	** *F* **^** *a* **^	**P**
**SEIQoL-DW (n = 91) (R**^**2**^ **= 13.9%)**				
**Number of psychotropic medications**		2.7	3.6	.063
		(−.14, 5.5)		
**CORE Non-risk score**		-.83	4.5	.038
		(−1.6, -.05)		
**Empowerment (n = 90) (R**^**2**^ **= 48.5%)**				
**Number of psychotropic medications**		−1.1	5.0	.027
		(−2.2, -.13)		
**Do you take this medication as prescribed?**	**Yes**	76.4	5.3	.007
		(74.7, 78.0)		
	**Partially**	71.3		
		(68.4, 74.2)		
	**No**	71.2		
		(65.2, 77.1)		
**Mental Health Confidence Scale (n = 90) (R**^**2**^ **= 46%)**				
**Age**		-.01	1.8	.178
		(−.02, .00)		
**Do you take this medication as prescribed?**	**Yes**	3.8	3.0	.056
		(3.6, 4.0)		
	**Partially**	3.4		
		(3.1, 3.7)		
	**No**	4.0		
		(3.4, 4.7)		
**Accommodation Status**	**Supported**	3.6	2.1	.156
		(3.4, 3.9)		
	**Unsupported**	3.9		
		(3.5, 4.2)		

^a^Analysis of covariance.

#### Predictors of engagement

In LO 48% of participants interviewed at follow up had a high level of engagement with the self-care intervention, in SO 63% and in NO 79%. Variables found to be univariately associated with being engaged with the self-care intervention were age, SEIQoL at T0, the STAR subscales of ‘positive collaboration’ and ‘positive clinician input’ at the 10% level. The two subscales were highly correlated, therefore STAR ‘positive collaboration’ was included in the model as the univariate association with engagement was stronger and felt to be more relevant in this context. Participants with a higher quality of life at T0 and who rated the STAR ‘positive collaboration’ subscale higher were more likely to stay engaged with the self-care intervention (OR = 1.02: 95% CI 1.00, 1.05; OR = 1.13: 95% CI 1.02, 1.26, respectively). Age was not significant in the combined model.

### Results - qualitative study

We present here key findings from our synthesis of qualitative and quantitative data as well as important overarching themes derived from the qualitative data set as a whole (the second and third stages of our analysis process). The full process and our descriptive analytical framework are presented in the full project report^1^. Our analysis suggested that study participants articulated their experiences and understandings of support for self-care in mental health on two levels: interviewees shared their experiences and thoughts about the components, structures and processes of support for self-care; interviewees were also concerned about the way in which that support was provided, identifying important qualities of supporting self-care in mental health. Presentation of our qualitative analysis below reflects both those dimensions; the components and qualities of supporting self-care in mental health. It is important to note that we did analyse data by site, but did not find any patterns of difference between sites (although more London participants talked about processes within the peer group, possibly because that was such an explicit component of that project). We indicate which site each quote originates from.

#### Taking control

Quantitative analysis showed improvement in levels of empowerment and mental health confidence over the course of nine months. Qualitative accounts indicated that participants specifically attributed improvement in related outcomes to their experiences of self-care support:

"It’s made me feel that I’m more competent with myself than I thought.’ (SO)"

"‘It’s helped me be more confident with my psychiatrist.’ (LO)"

"‘It’s made me feel more stronger and I’ve got more self esteem ‘cos I know I can put things to the test.’ (NO)"

These findings reflect an understanding of self-care that is about taking, or regaining control:

"‘… it’s taking sort of charge a little bit, I think a lot of the sort of problems are you are sort of losing control, a lack of control, suddenly you feel you’re completely out of control and the self-help is getting back in control…’ (SO)"

This sense of timing engagement in support for self-care was seen as essential to participants

"‘When the time is right, yes. I've got no doubt I will do it, it is just a question of when the time is right to do it.’ (SO)"

"‘It would be dangerous to try and force myself if I was really ill to do it’ (NO)"

Many participants referred to the importance of being in control of when and how they made use of support for self-care, and genuine opportunity to self-refer was identified as a facilitator of having that control:

"‘I was told it was a self referral project as opposed to something that you are referred to, the onus is on you to go yourself … it’s your control whether you go or not … I didn’t want to go because someone had told me to go, I wanted to go because I wanted to go in itself.’ (LO)"

Having flexibility of ongoing access to support, when and how the individual found it necessary was valued, and contrasted with experiences of punitive discharge for non-attendance of other mental health services:

"‘That’s a recognition of the way that people’s problems work, is that they may turn up for a while then not turn up for a while and … they don’t exclude you because of that.’ (LO)"

"‘… if you got sick and didn’t turn up twice you got discharged. You can’t help it if you’re sick.’ (SO)"

*Personal plans* – Developing a personal plan was also seen as a facilitator of taking control:

"‘Whereas a care plan is basically, this is what is going to happen, with a WRAP [personal plan], it is basically you taking control, and you taking responsibility for what happens to you.’ (SO)"

"*Peer groups –* Many participants reported the benefits of peer group based support:"

"‘I could leave a group at the … project feeling more positive, simply from what I felt I did for someone else.’ (LO)"

Peer groups provided opportunities to share knowledge about coping and self-care strategies:

"‘… other people will be there so they can sort of like shed fresh light onto how you can best cope with it … Usually they tend to be going through the same situation as well, so we can talk like, share experiences.’ (LO)"

Peer groups also provided a site for nurturing ‘well’ identities, especially where groups took place in community settings:

"‘You never feel as if you’re attending something that is to do with mental health, that was what I found, which feels like you are taking more part in the normal world outside.’ (NO)"

"‘… it’s made me more sociable, less isolated, things like that … The main thing was, is that it centres around the real world.’ (LO)"

However, groups could be challenging for participants on a number of levels, especially where self-referral meant that groups were open access:

"‘I just don't like to hear the arguments and the clash of personalities there and that kind of puts me off as well. I think “blimey I don't need to be around this”. So there've been a couple of times when I haven't gone because of that.’ (LO)"

"‘I was still worried about confidentiality, because speaking about yourself in a group where maybe you don’t know the people very well, you don’t know if they would quite casually mention the things that you hoped would be confidential.’ (SO)"

"‘… there were times when some people, I just took an instant dislike to and, but they were new and I didn’t know them, so I just couldn’t talk.’ (LO)"

While peer groups provided the benefits of routine and structure, for some participants they also created new sites of dependence that could act as barriers to self-care:

"‘… it’s something I shouldn’t do but I’m starting to depend on the place a bit and I think that’s a bad thing really. I shouldn’t depend on it but I am, I can’t help it, it’s because I feel kinda at home here.’ (NO)"

"‘… the people that went there went there for their social life … but it becomes your identity … you don’t feel left out, you’ve all got a common bond ‘cos you’re all mental, in one regard or another. And it’s comfortable and it’s supportive. Of course you never get better, I don’t think, because of that, I think that’s the only problem’. (LO)"

#### Staff-service user relationships

Statistical analysis demonstrated that there was some association between strong positive collaboration between participant and the member of staff on the self-care project with whom they had the most contact, and a higher level of engagement with the project. Qualitative accounts complemented this finding, indicating the importance of relaxed, supportive relationships between service users and staff:

"‘They support you but they are not actually looking over your shoulder, it’s not an uncomfortable type of support and I think that is how it works, you know, that’s why it works so well for me I think, it’s relaxed but directed as well if you know what I mean.’ (NO)"

"‘… they are not judgemental and they have been so supportive … it feels less formal, they seem very caring … it's less formal than with doctors or occupational therapists … it just seems more of a genuine relationship.’ (NO)"

"‘… their attitude is really good … they’ve got insight into what it might be like for me coming along to a new group on the day and being a bit nervous about joining in and that, very understanding.’ (NO)"

However, it was also noted how some participants found the approach too relaxed, or noted the need for staff to be more hands on in their support where need arose:

"‘Actually that’s one of the things that perhaps wasn’t done as professionally as it could have been … It was all a bit low key and a bit laid back, and a bit … sometimes almost being wishy-washy really … Not enough direction.’ (SO)"

"‘The need to be supportive can run into a little bit of trouble and then again that's where the staff come in more, to sort of like, I don't know, sort of like emergency brakes or a sort of, just as a sort of non-invasive safety net …’ (LO)"

All self-care initiatives included service users employed in co-facilitation roles, and participants commented on the benefits and challenges of the role:

"‘I particularly liked and warmed to the fact that [the service user trainer], the leader was not, that it wasn't an 'us and them'. I think that really impressed me, that she had been there done that and bought the T-shirt and that she was open and honest enough to share it…’ (SO)"

"‘Well I think they had more baggage than we had really. Sort of working through things when we should have been … so it was taking away from the patients really.’ (SO)"

*Medication and supporting self-care -* We identified, quantitatively, a number of medication variables at baseline that were significantly associated with outcome at follow up. It was possible to further explore those associations by running a series of queries on the qualitative data. Many participants who chose to take their medication as it was prescribed viewed medication as being part of their overall self-care:

"‘Giving [medication] a go again and making sure that I commit to it, sort of feels like self-care …’ (LO)"

"‘…with me it took a long take to get my medication sorted out and once you’re on a stable level with that then you can start caring for yourself’ (SO)"

Many participants who chose not to take their medication, however, talked about the importance of self-care coming from the individual rather than from medication:

"‘… trying to get better on my own without the help of medication…’ (NO)"

"‘ try not to rely on my medication … in the end the only way to make myself well was to confront my fears a bit and not rely on it…’ (SO)"

## Discussion

### Implementing generic self-care policy in the mental health context

We set out to investigate the implementation of generic UK self-care policy in the context of mental health services for severe, long term mental health problems. Our quantitative findings did demonstrate improvements in some of the self-care outcomes indicated in the policy literature [5,6] during the nine months of the study. A reduction in the use of A&E for psychiatric emergency indicates potential cost benefits of supporting self-care [43]. Our findings also indicated improvements in Empowerment, Mental Health Confidence and Quality of Life. Although improvements were statistically modest these reflected findings elsewhere for guided self-help for depression [44,45] and self-management programmes in general [11]. It should also be noted that the mean length of contact with services of our sample – typical users of secondary mental health services – was nearly fifteen years. It has been argued that, in the management of long term conditions, improvements that facilitate independent living and the engagement of the patient in their own care are more important than the more dramatic improvements associated in acute care with clinical cure [46]. The importance to service user participants of improvements in Empowerment and Confidence was clearly indicated in our qualitative data and, in many cases, attributed by individuals to the support they were receiving in the self-care initiatives.

As this was an observational study we cannot, methodologically, attribute these changes in outcome directly to the case study initiatives. However, these findings both demonstrate for service providers that interventions supporting self-care in mental health are likely to impact on those outcomes anticipated in the policy guidance, and would enable selection of outcome measures and sample size calculations for future controlled studies.

Qualitative findings also indicated that study participants, on the whole, valued service delivery components advocated in the policy literature: peer support groups; personal planning initiatives; sharing of information and coping strategies; ‘lay-led’ support; support provided in community settings. A range of benefits were attributed to peer support groups in particular, including opportunities to share coping and self-care stategies, develop ‘well’ identities and build social networks. However, our qualitative analysis also indicated difficulties with peer support groups, such as anxieties about joining groups and dependency. Our data suggested that service users working in the delivery of self-care support (peer workers) were valued, although concerns were expressed about managing the mental health of peer workers. A literature is emerging that seeks to understand the implementation issues – as well as benefits and challenges – of employing peers in the delivery of mental health services [47].

### Understanding the mental health specific issues of supporting self-care

Our exploratory quantitative analysis indicated a lack of association, across sites, between level of engagement with specific components self-care support and outcome. Elsewhere it has been shown that mode of delivery is not associated with outcome in guided self-help interventions for depression [44]. Qualitative data indicated the importance for service users of having control over when and how they used services supporting self-care. Together these findings suggest that support for self-care cannot be ‘dosed’; that prescribing a minimum attendance at peer support groups, for example, does not equal a better ‘treatment effect’. The implications of this for providing support for self-care in mental health include the need to better understand the dynamics and difficulties of self referral into services [48] and to move away from a culture of punitive discharge from services following non attendance [49].

Our exploratory analysis also indicated some association between high participant ratings of ‘positive collaboration’ with intervention staff and high levels of engagement with interventions. Qualitative accounts indicated the importance for service users of non-judgmental, relaxed service user-staff relationships. It should be noted, however, that service users were concerned where they felt that that support was too relaxed, especially should they experience a crisis in their mental health. These findings reflect recent research exploring the role of the mental health professional in enabling and facilitating a more patient directed care [50-52].

The complexity of findings around medication was further indicative of mental health specific issues of supporting self-care. Synthesis of quantitative and qualitative analyses suggested that participants who had taken a decided course of action on whether or not to take their medication did so as part of their self-care. This reflects recent thinking about the importance of shared decision making about medication by service users and clinicians [53,54], ongoing debates around issues of treatment concordance and adherence [51,55], as well as research that links therapeutic relationship with medication adherence [56].

### Generalising findings across mental health settings

In this study we deliberately selected contrasting cases in order to consider the extent to which our observations applied across mental health settings. The resulting heterogeneity of our sample was our biggest methodological challenge. Most change in outcome was observed in the London site, where the sample was younger, broadly ‘less well’, and where the intervention was designed to support people experiencing personality disorders. High levels of service use have been shown in populations where Personality Disorders are comorbid with other psychiatric disorders and substance misuse [57], as was the case with our sample, and studies in other fields have indicated how response to intervention can be better where initial clinical severity is higher [58,59]. In seeking to demonstrate the effectiveness of interventions supporting self-care in mental health, future experimental studies should be careful to limit heterogeneity in the sample. In addition, as one of our study sites was unable to quantify the number of potential recipients of the intervention (see Figure 2) we were unable to estimate the representativeness of the sample in that site. This limits the extent to which we can generalise specific findings across mental health populations.

However, our inclusive approach to case study selection did lend a broad external validity to our findings. Our qualitative findings were similar across sites, irrespective of the different structure or conceptual underpinning of the initiatives, and of differences in the sample. In particular the issue of having control over when and how to access support for self-care was important to service users in all settings. Our core results, quantitative and qualitative, stood up across case studies, suggesting that findings are broadly generalisble across mental health settings.

## Conclusions

This study has sought to inform the development and delivery of services supporting self-care for people with severe, long term mental health problems. We have demonstrated that self-care is supported both by the provision of a range of service delivery components – peer support groups, personal planning initiatives, ‘lay-led’ support – and through an approach to supporting self-care that is informed by a number of key mental health specific issues. These issues included giving the service user control over when and how support to self-care is accessed, and new staff-service user relationships characterised by a less directive, enabling approach. A change in the culture of mental health care is implicit in these expectations, and further organisational research is needed to understand how care management and role changes of this sort are introduced by mental health service providers. The broad empirical basis of our research indicates the wide relevance of our findings across mental health settings and service delivery models, while also providing insight into the methodological challenges of undertaking experimental research to establish the effectiveness of interventions supporting self-care in mental health.

## Competing interests

The author(s) declare that they have no competing interests.

## Authors’ contributions

SG was principal investigator and led on the design, implementation and writing up of the research. KA undertook data collection and analysis, contributed to writing up the research and brought a carer’s perspective to the project. CE led on the organisational component of the study, contributing to study design, analysis and writing up the research. ML led the North of England study site and contributed to analysis and checking drafts of the manuscript. SM led the London study site, provided clinical oversight for the study, provided guidance on writing up the research and checked drafts of the manuscript. LS led the South of England site, supported service user researcher involvement in the study and contributed to analysis and writing up the research. KT brought a service user researcher perspective to study design, data collection, analysis and writing. RW undertook data collection and analysis, and contributed to writing up the research. SW developed and undertook the statistical analysis for the research and led on writing up that analysis. All authors read and approval the final manuscript.

## UK department of health disclaimer

The views and opinions expressed therein are those of the authors and do not necessarily reflect those of the SDO programme, NIHR, NHS or the Department of Health.

## Pre-publication history

The pre-publication history for this paper can be accessed here:

http://www.biomedcentral.com/1472-6963/12/189/prepub
